# An Unusual Case of Diffuse Alveolar Hemorrhage as a Clinical Manifestation of Atypical Hemolytic Uremic Syndrome: A Case Report

**DOI:** 10.7759/cureus.5059

**Published:** 2019-07-01

**Authors:** Ahmad Al-Shyoukh, Moustafa Younis, Omar Abughanimeh, Mohammad Tahboub, Majdi S Hamarshi

**Affiliations:** 1 Internal Medicine, University of Missouri-Kansas City l Saint Luke's Health System, Kansas City, USA; 2 Critical Care, University of Missouri-Kansas City l Saint Luke's Health System, Kansas City, USA

**Keywords:** hemolytic uremic syndrome, atypical hemolytic uremic syndrome, diffuse alveolar hemorrhage, eculizumab, campylobacter jejuni

## Abstract

Hemolytic uremic syndrome (HUS) is a constellation of microangiopathic hemolytic anemia, thrombocytopenia, and acute renal injury. HUS is subcategorized into primary or secondary HUS. Primary HUS is synonymous with atypical HUS (aHUS) and is attributed to genetic complement deficiency. Diffuse alveolar hemorrhage (DAH) is a serious condition complicating multiple systemic conditions. aHUS presenting as DAH is exceedingly rare. In this case, we present a 75-year-old male patient who presented with generalized weakness, malaise, and hemoptysis. He was found to have hemolytic anemia and thrombocytopenia, with elevated creatinine. Bronchoscopy confirmed DAH. He was started on plasmapheresis with a suboptimal response. aHUS was suspected and the patient was started on eculizumab with subsequent laboratory and clinical improvement. HUS and aHUS can present as DAH. It is very important to recognize both conditions as both are life threatening with high morbidity and mortality.

## Introduction

Hemolytic uremic syndrome (HUS) is a condition that falls under the umbrella of thrombotic microangiopathies (TMA). It presents with microangiopathic hemolytic anemia, thrombocytopenia, and acute renal impairment [1-2]. HUS is traditionally classified into diarrhea positive or typical HUS that is preceded by certain infections such as Shiga-toxin-producing *Escherichia coli* (STEC) and atypical HUS (aHUS) that is attributed to genetic dysregulation of the alternative complement pathway [2-3]. Previously, aHUS was also used to refer to any HUS case that was not caused by STEC. More recently, HUS is classified as either primary HUS, synonymous with aHUS and complement-mediated HUS, which is due to genetic alterations of the alternative complement pathway or secondary HUS that is attributed to infections, malignancy, autoimmune conditions, hypertensive emergencies, pregnancy or drugs [4]. Though aHUS predominantly presents in the pediatric population, it has also been identified in adults [4-5]. Though unknown in the adult population, the estimated prevalence of complement-mediated aHUS in the pediatric population is seven per one million [6].

Diffuse alveolar hemorrhage (DAH) is a life-threatening syndrome that presents with hemoptysis, anemia, hypoxia and diffuse alveolar infiltrates [7-8]. A variety of diseases, including hematological, infectious, or immunological etiologies, are associated with the development of DAH. Idiopathic thrombocytopenic purpura and thrombotic thrombocytopenic purpura (TTP) are well-known causes of DAH resulting from vasculitis or capillaritis [8]. However, HUS and specifically aHUS manifesting as DAH is exceedingly rare. It is important to distinguish aHUS from other TMA as management and prognosis vary [3]. The mainstay of treatment is supportive therapy along with eculizumab, a monoclonal recombinant antibody that inhibits complement factor five, which has been proven to treat and prevent recurrences of aHUS [9]. Herein, we illustrate the morbidity and management of a rare case of *Campylobacter* gastroenteritis triggering aHUS that presented with DAH.

## Case presentation

A 75-year-old male with a past medical history of rheumatoid arthritis, controlled on methotrexate and adalimumab, presented to an outside hospital with diarrhea, intermittent bright red blood with stool, and decreased oral intake. He was hypotensive (70/46 mm Hg) and tachycardic at 103 beats per minute (bpm). The rest of his vitals were otherwise unremarkable. His physical exam was only remarkable for mild right upper quadrant abdominal tenderness. His labs were significant for an elevated white blood cell (WBC) count of 22.5 x 103/mm^3^ and an acute kidney injury (AKI) with a serum creatinine of 1.83 mg/dl. His hemoglobin, platelet count, electrolytes, and liver function tests were all within normal limits. The patient was admitted to the floor for further management. He was started on intravenous (IV) fluids and completed a course of azithromycin with significant improvement in his symptoms. His polymerase chain reaction (PCR) stool studies were eventually positive for *Campylobacter jejuni*. On hospital day four, he was discharged. Upon discharge, his hemoglobin was 10.6 g/dl, platelets were 236 x 10^3^/mm^3^, WBC count was 11.7 x 10^3^/mm^3^ and creatinine was 0.90 mg/dl.

Five days later, our patient presented to our institution with progressively worsening generalized weakness, fatigue, nausea, vomiting, intermittent shortness of breath and hemoptysis. The hemoptysis was small in amount, bright red in color without blood clots. He denied any chest pain, fever, or chills. His presenting vital signs were a heart rate of 96 bpm, blood pressure (BP) of 136/69 mm Hg, and oxygen saturation of 94% on room air. His physical exam was noteworthy for conjunctival pallor, scattered ecchymosis and petechia and bilateral lower extremity edema. Patient’s labs were significant for a drop in his hemoglobin and platelet count to 7.1 g/dl and 56 x10^3^/mm^3^, respectively. His reticulocyte count was elevated at 9.2% along with an elevated lactate dehydrogenase level (5454 U/L), a low haptoglobin level (< 10 mg/dl), and an elevated total and indirect bilirubin of 2.2 mg/dl depicting acute hemolytic anemia. The patient also demonstrated worsening renal function with a blood urea nitrogen of 66 mg/dl and serum creatinine of 3 mg/dl. He had a normal international normalized ratio (INR) and elevated fibrinogen level (451 mg/dl). His chest X-ray showed bilateral pulmonary opacities mostly within lung bases. Given the concern for microangiopathic hemolytic anemia and multiorgan failure, he was admitted to the intensive care unit (ICU). Schistocytes were visualized on his peripheral smear. The hematology and oncology team was consulted and plasmapheresis was initiated given the possibility of TTP.

**Figure 1:**
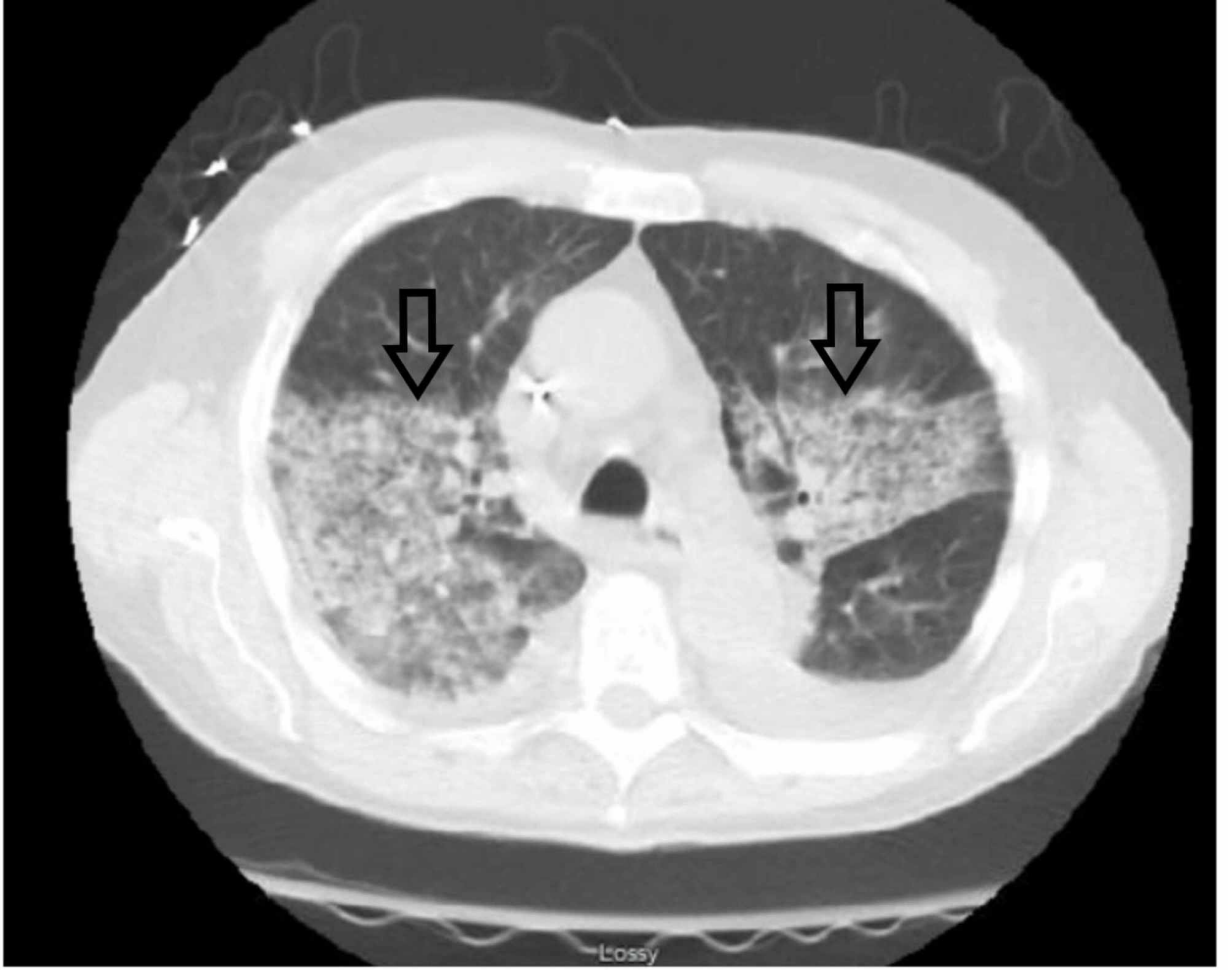
Computed tomography scan of the chest without contrast demonstrating bilateral perihilar heterogeneous opacities

On hospital day (HD) two, the patient developed acute hypoxic respiratory failure with oxygen saturations as low as 86% on room air requiring oxygen supplementation with the nasal cannula at 3 liters/minute. Worsening bilateral perihilar heterogenous opacities were observed on the patient’s CT scan of the chest without contrast (Figure 1). He was started empirically on vancomycin, piperacillin-tazobactam, and ciprofloxacin. Flexible bronchoscopy with sequential bronchoalveolar lavage showed that lavage aliquots are progressively more hemorrhagic, highly suggestive of DAH. On HD three, the patient was started on methylprednisolone 62.5 mg every 6 hours, due to concerns for vasculitis. Further workup revealed negative antineutrophil antibody (ANA), antineutrophil cytoplasmic antibody (ANCA), anti-glomerular basement membrane (Anti-GBM), and stool Shiga toxin one and two. Complement levels of C3, C4, and ADAMTS13 (a disintegrin and metalloprotease with thrombospondin type 1 repeats-13) activity level (90%) were within normal limits. 

The patient underwent a total of eight sessions of plasmapheresis and received multiple blood transfusions with minimal improvement in his hemoglobin and platelet count. Subsequently, atypical HUS labs screening for genetic mutations and antibodies to complement factors were sent and he was started on eculizumab 900 mg intravenously once weekly. His methylprednisolone was switched to an oral prednisone taper. He was discharged on HD 16 after his second dose of eculizumab. The patient was discharged home to continue eculizumab 900 mg IV once weekly as an outpatient. He received two more doses of eculizumab 900 mg IV and it was switched to eculizumab 1200 mg once every two weeks, which patient continues to receive to date. During his two months follow up, the patient’s clinical condition and laboratory findings significantly improved (Table 1). Further, his final aHUS workup that was drawn after plasmapheresis was unremarkable with the exception of a low factor H complement antigen 21.6 mg/dl (normal range: 23.6-43.1) and a high SC5b-9 complement 330ng/ml (normal range <251).

**Table 1:** Laboratory values

Significant event	Hospital Day	Hemoglobin (g/dl)	Hematocrit (%)	Platelet count x10^3^/mm3	Lactate dehydrogenase level (U/L)	Creatinine (mg/dl)
Plasmapheresis initiated	Day 1	6.4	19	67	5535	3.1
	Day 2	7.6	21	88	2351	2.8
	Day 3	8.1	23	147	1843	3.2
	Day 4	6.6	19	130	1091	3.1
	Day 5	7.5	22	98	1024	2.9
	Day 6	6.9	21	86	1105	2.6
	Day 7	7.4	22	83	1071	2.7
	Day 8	7.5	22	77	1067	2.5
Eculizumab initiated	Day 9	7.3	22	80	1080	2.3
	Day 10	7.5	22	91	1614	2.5
	Day 11	7.1	21	92	1899	2.4
	Day 12	7.1	22	104	2051	2.2
	Day 13	7.5	23	104	2228	2.1
	Day 14	7.3	22	131	2120	2.1
	Day 15	6.7	20	122	1988	2.1
Discharge	Day 16	7.5	22	128	1955	2.2
	Day 60	12.4	37	274	670	1.8
	Day 150	14	41	241	453	1.4

## Discussion

We present a rare case of a 75-year-old male patient who came in with generalized weakness, fatigue, and hemoptysis preceded by an episode of *Campylobacter* diarrhea. It was associated with thrombotic microangiopathy and diffuse alveolar hemorrhage confirmed by bronchoscopy. He was managed with plasmapheresis initially without significant clinical improvement, later necessitating treatment with eculizumab. The presentation is significantly rare, and to the best of our knowledge, there are only two reported cases of *Campylobacter*-associated HUS associated with pulmonary hemorrhage documented in English literature [10-11].

aHUS refers to HUS cases due to genetic or acquired dysregulation of the alternative complement system pathway and coagulation cascade [1-2]. It is also referred to as primary or complement-mediated HUS. HUS results from endothelial cell injury, mainly in the renal microvasculature, resulting in the formation of fibrin and platelet thrombi. This injury can result from several mechanisms depending on the etiology of the inciting event. The formation of these thrombi leads to shearing of red blood cells and consequently the formation of schistocytes. Thrombocytopenia results from consumption of platelets in thrombi formation [12]. In aHUS, genetic or acquired dysregulation of the alternative pathway of the complement cascade in addition to an external trigger, such as *Campylobacter* in our case, leads to the formation of membrane attack complex C5b-9, which binds to endothelial cells’ surface causing cell damage and swelling, thus creating a nidus for thrombi formation [2,12]. Either autoantibodies against Factor H or mutations in genes coding complement regulatory proteins, such as Factor H (CFH), Factor I (CFI), membrane cofactor protein (MCP), complement 3 (C3), FactorB (FB), or thrombomodulin contribute to this dysregulation [9]. This highlights the role of eculizumab in the management of aHUS: a monoclonal antibody that binds to complement protein C5 and then blocks the formation of C5 convertase, which ultimately leads to inhibition of the alternative complement pathway. aHUS cases are usually triggered by an infection. Keithlin et al. performed a metanalysis reviewing chronic sequelae of *Campylobacter jejuni* infection, with three out of the 31 studies reporting on the incidence of *Campylobacter*-induced HUS [13]. The first study encompassing a pediatric population did not have any reported cases of HUS, while the other two surveillance-based studies reported estimates of 0.005% and 0.003% [13].

aHUS can present at any age [5]. A study done in France by Fremeaux-Bacchi et al. evaluating 214 patients with aHUS showed that 58.4% of the affected individuals were adults [14]. Patients with HUS present with non-specific symptoms such as fatigue, malaise, shortness of breath, anorexia, decreased urine output and edema [1-2]. There have been some reported cases of aHUS presenting as DAH, but more data is needed to estimate the actual incidence [15]. In our case, and in other similar reported cases, it is unclear what is the exact mechanism of diffuse alveolar hemorrhage in patients with HUS; however, thrombocytopenia and thrombi formation in pulmonary vasculature could be contributing factors. 

The diagnosis of aHUS is challenging as it resembles other TMA conditions including TTP. The diagnosis is usually suspected when patients present with the triad of microangiopathic hemolytic anemia, thrombocytopenia and acute renal injury [9]. In our patient, after recognizing a TMA, the first step was to exclude TTP. The distinction between HUS and TTP can be done based on ADAMTS13 activity [2]. However, ADAMTS13 levels are not usually available at the time of decision making thus empiric management, such as in our case, with plasmapheresis is vital. Also, the diagnosis of aHUS is mostly done after the exclusion of other TMA conditions [5]. We subsequently attempted to rule out Shiga toxin-mediated HUS and other potential secondary etiologies that might trigger HUS. Despite the first sessions of plasmapheresis, our patient’s platelet counts remained unchanged. Given the exclusion of secondary etiologies and the poor response to plasmapheresis; aHUS was highly suspected. The diagnosis of aHUS further requires the demonstration of either genetic mutations and/or antibodies to complement factors [16]. These tests are not widely available and take weeks to result [9]. Despite the lack of family history, we elected to proceed with further aHUS work up given the poor clinical course in the absence of an underlying etiology. In our patient, the low Factor H can be consistent with aHUS, however, with low C4 and high SC5b-9, We believe this is not reflective of what our patient had as these labs were drawn after multiple sessions of plasmapheresis [4].

HUS management is supportive including fluid and electrolyte replacement, blood transfusion and dialysis [1-2]. In aHUS, plasmapheresis was traditionally the mainstay of treatment. However, it does not address complement dysregulation [9]. Lack of clinical and laboratory response after five to seven sessions of plasmapheresis should warrant the initiation of eculizumab if available [9]. Studies have shown that eculizumab improves prognosis and survival [9]. The clinical improvement seen in response to eculizumab further confirms the diagnosis of aHUS. Testing for anti-CFH antibodies prior to eculizumab infusion is the only urgent complement investigation required, as plasmapheresis would be the treatment of choice in such cases [9]. Eculizumab is administered as an intravenous infusion with weight-based induction and maintenance dosing [9]. Eculizumab therapy can be discontinued in patients who have had a favorable clinical and laboratory response to therapy [9]. There is a paucity of data defining the optimal timing for infusion cessation; therefore, the decision to withdraw eculizumab therapy should be made in conjunction with a clinician with expertise in managing aHUS. Finally, the prognosis in aHUS is not favorable with a mortality rate of 25% during the acute phase [3,9]. Around, 50% of the patients may progress to the end-stage renal disease within a year [3,9].

## Conclusions

In conclusion, aHUS triggered by *Campylobacter* gastroenteritis presenting as DAH is rare and life-threatening. There have been some reported cases of aHUS presenting as DAH, but more data is needed to estimate the actual incidence. A high degree of clinical suspicion is necessary to establish diagnosis after ruling out other etiologies of TMA such as TTP. In comparison with other TMAs, management of aHUS with eculizumab is unique highlighting the importance of proper diagnosis. It is encouraged to report such cases and highlight the management to help set up the stage to formulate a unified consensus for treatment.
